# Bone Marrow-Derived Stem Cell Populations Are Differentially Regulated by Thyroid or/and Ovarian Hormone Loss

**DOI:** 10.3390/ijms18102139

**Published:** 2017-10-19

**Authors:** Bassam F. Mogharbel, Eltyeb Abdelwahid, Ana C. Irioda, Julio C. Francisco, Rossana B. Simeoni, Daiany de Souza, Carolina M. C. O. de Souza, Míriam P. Beltrame, Reginaldo J. Ferreira, Luiz C. Guarita-Souza, Katherine A. T. de Carvalho

**Affiliations:** 1 Cell Therapy and Biotechnology in Regenerative Medicine Department, The Pelé Pequeno Príncipe Institute, Child and Adolescent Health Research & Pequeno Príncipe Faculty, Ave. Silva Jardim 1632, Box 80.250-200 Curitiba, Paraná, Brazil; bassamfm@gmail.com (B.F.M.); anairioda@gmail.com (A.C.I.); julio.apfr@gmail.com (J.C.F.); daianys.bio@gmail.com (D.d.S.); carolina.souza@fpp.edu.br (C.M.C.O.d.S.); reginaldojf@hotmail.com (R.J.F.); 2 Feinberg School of Medicine, Feinberg Cardiovascular Research Institute, Northwestern University, 303 E. Chicago Ave., Tarry 14-725, Chicago, IL 60611, USA; eltyeb.abdelwahid@northwestern.edu; 3 Experimental Laboratory of Institute of Biological and Health Sciences of Pontifical Catholic University of Paraná (PUCPR), Rua Imaculada Conceição, 1155, Box 80.215-901 Curitiba, Paraná, Brazil; rossanab@tecpar.br (R.B.S.); guaritasouzalc@hotmail.com (L.C.G.-S.); 4 Cytometric Analysis. Hospital de Clínicas, Haematology Department, Federal University of Paraná, Av. General Carneiro, 181-Box 85.002-490 Curitiba, Paraná, Brazil; mbeltrame@ufpr.br

**Keywords:** hypothyroidism, estrogen, precursor B cells/Pro-B cell, mesenchymal stem cell, endothelial progenitor cell, bone marrow, ovariectomy, thyroidectomy

## Abstract

Bone marrow-derived stem cells (BMDSCs) play an essential role in organ repair and regeneration. The molecular mechanisms by which hormones control BMDSCs proliferation and differentiation are unclear. Our aim in this study was to investigate how a lack of ovarian or/and thyroid hormones affects stem cell number in bone marrow lineage. To examine the effect of thyroid or/and ovarian hormones on the proliferative activity of BMDSCs, we removed the thyroid or/and the ovaries of adult female rats. An absence of ovarian and thyroid hormones was confirmed by Pap staining and Thyroid Stimulating Hormone (TSH) measurement, respectively. To obtain the stem cells from the bone marrow, we punctured the iliac crest, and aspirated and isolated cells by using a density gradient. Specific markers were used by cytometry to identify the different BMDSCs types: endothelial progenitor cells (EPCs), precursor B cells/pro-B cells, and mesenchymal stem cells (MSCs). Interestingly, our results showed that hypothyroidism caused a significant increase in the percentage of EPCs, whereas a lack of ovarian hormones significantly increased the precursor B cells/pro-B cells. Moreover, the removal of both glands led to increased MSCs. In conclusion, both ovarian and thyroid hormones appear to have key and diverse roles in regulating the proliferation of cells populations of the bone marrow.

## 1. Introduction

The transplantation of stem cells has been proposed as a novel approach for the treatment of patients with several diseases, including ischemic heart injury [1]. Various cells, including bone marrow derived as mononuclear cells, endothelial progenitor cells, mesenchymal stem cells (MSCs), and skeletal myoblasts, have been examined in ischemic heart models [1,2,3,4]. A major issue with this promising therapy is the proper vascularization and maintenance of an adequate number of surviving cells after cell transplantation [5,6,7,8,9,10] Tissue replacement has been proposed to repair congenital defects or diseased tissue [1,2,3,4]. Endothelial progenitor cells (EPCs) of bone marrow have been proposed to have potential therapeutic applications for ischemic cardiovascular diseases [5,6,7,8]. Bone marrow-derived EPCs are known to circulate to neovascularization sites where they differentiate into endothelial cells and contribute to vasculogenesis [9,11,12] and the neovascularization of ischemic injuries [5,6,7,8]. Stem cells derived from bone marrow have distinct and specific functions: hematopoietic stem cells, EPCs, and non-hematopoietic stem cells [13]. While hematopoietic stem cells give rise to blood components, EPCs secrete various endothelial growth factors, promoting angiogenesis in pathological conditions [14]. Non-hematopoietic stem cells as MSCs can differentiate to myogenic, osteogenic, chondrogenic, and adipogenicmesodermal lineages. Moreover, the MSCs are able to differentiate into ectodermal tissues, such as glial cells [15,16]. One way to identify hematopoietic, endothelial, and mesenchymal stem cells is through their membrane markers. Hematopoietic stem cells exhibit the membrane markers CD45+ and CD34+. While endothelial cells are phenotypically identified by CD34+ and CD45−, MSC show the CD45− and CD34− phenotypes [17]. In addition, there are other cells that are also recognized by the CD45− and CD34+ phenotypes, e.g., platelets and cells oferythroid lineage; however, the EPCs can be differentiated by specific markers, such as CD31+.

Thyroid and ovarian hormones regulate cellular homeostasis, proliferation, apoptosis, and differentiation [18]. Investigating the hormonal regulation of stem cells allows for an understanding of stem/progenitor cell physiological and biological activities that are required for important therapeutic applications. The role of thyroid and ovarian hormones in stem cell function is still unclear. Thyroid hormones have been shown to inhibit the proliferation of some cells, including cultured embryonic neural stem cells, and have been suggested to regulate the activity of insulin-like growth factor type 1 (IGF-1) [19,20]; however, their roles in the proliferation of bone marrow stem cells have not been clarified [21]. Previous investigators have reported that ovariectomy upregulates B lymphopoiesis in rat bone marrow and that decreased estrogen is involved in the expansion of B cell precursor and mature B cell populations; however, the role of B lineage cells in ovariectomy-induced bone loss in vivo is unclear [22,23]. It was previously reported that there are bidirectional interactions between the hypothalamic-pituitary-thyroid (HPT) axis and the immune system [24]. While hypothyroidism leads to an inhibition of humoral and cell-mediated immune responses, hyperthyroidism was found to upregulate immunity.

The formation of new blood vessels is essential for successful bone marrow stem cell transplantation and tissue regeneration [25]. On the other hand, the proliferation, differentiation, and survival of progenitor populations depends on various factors secreted by other cell types. Specifically, a wide range of secreted factors regulates the proliferation and fate of stem cells; however, the mechanisms have not been identified. Hormonal action is regulated by multiple signaling molecules that control vital biological processes of various body cell types, such as growth, development, proliferation, and differentiation. The end result of hormonal action is a general increase in functional activity throughout mammalian cells, including cell proliferation [26]. Thyroid hormones can act indirectly to regulate the transcription of genes that influence metabolism, cell growth, and the development of all organ systems. Therefore, the clinical manifestation of thyroid disturbances are typically insidious, multiple, and diverse. The main form of thyroid hormone secreted by the thyroid gland is 3,5,3′,5′-tetraiodo-L-thyronine (T4 or thyroxine). The thyroid gland also secretes a smaller quantity of 3,5,3′-triiodothyronine (T3), the active form of the hormone. In addition, the concentration of T4 is about 45 times higher than that of T3 (90 nm versus 2 nm), and the primary production source of T3 occurs by the conversion of T4 to T3 in peripheral tissues [27]. Besides, the actions of these hormones require receptors in both the cell membrane and the nucleus [28]. In the presence of these hormones, thyroid hormone receptors bind with co-activator protein and allow for the organization and activation of transcription machinery [29]. Hypothyroidism is the most common pathology of the thyroid gland. In most cases, it is caused by abnormalities of the thyroid gland itself (primary hypothyroidism), such as, not producing enough of the hormone. Thyroid hormone level has been suggested to modulate the level of critical cell cycle proteins, specifically cyclin A, resulting in an alteration of S-phase progression [30]. Moreover, the action of pituitary leptin as a local inhibitor of Thyroid Stimulating Hormone (TSH) release also disappears in hypothyroidism [31].

Ovarian estrogens regulate the proliferation and growth of sex organs and other tissues related to reproduction, but their role is not limited to reproductive functions. Estrogens also play an important role in the balance between bone formation and resorption, and their loss causes critical physiological and pathological problems [32].

Given the above-mentioned facts, there is a growing interest to study hormonal influence on the proliferation of stem cells, especially the role of the thyroid and ovarian hormones in females. Many patients are already being subjected to bone marrow samples, with the purpose of isolating hematopoietic stem cells from bone marrow, without prior knowledge of the proliferative state of the cells to be transplanted. Increased or decreased stem cell proliferation maybe a significant factor in determining an efficient therapeutic result. To ensure greater success in transplantation, the integration of transplanted stem cells in vivo or ex vivo is favored by the increased proliferative capacity of the stem cells. In this study, we analyzed proliferative activity by detecting bromodeoxyuridine (BRDU), which is incorporated by cells during the S-phase of mitosis. This marker does not require immunohistochemistry and can be quantified by flow cytometry [33].

In the present study, we used bone marrow-derived mesenchymal stem cells, which have been reported to be very efficient in regenerative medicine due to their multi-lineage differentiation character and high potential to repair tissues. We compared the proliferative activity of these cells with other bone marrow-derived stem cells (BMDSCs) of rats subjected to the same conditions (ovariectomy and/or thyroidectomy). This enabled us to analyze the role of hormonal interactions (in vivo) in regulating BMDSCs expansion. We used specific markers and flow cytometry to assess the response of affected cell populations. Our results revealed diverse effects of thyroid and ovarian hormonal loss on BMDSCs proliferation.

## 2. Results

### 2.1. Surgeries’ Efficacy Confirmation Using Pap Smear and Thyroid Stimulating Hormone Levels

#### Pap Smear

**Figure 1:**
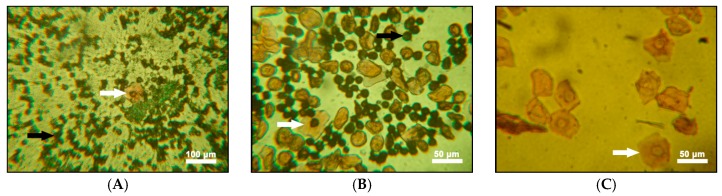
Vaginal swab samples stained with Pap. (**A**) Sample from a vaginal swab of a thyroidectomized female Wistar rat. Note the large number of lymphocytes (black arrow) characterizing the metestrus stage and rarely epithelial cells (white arrow); (**B**) A smear of a sample from an ovariectomized animal. In the diestrus stage, there is a large number of lymphocytes (black arrow) in addition to some epithelial cells (white arrow); and (**C**) In the proestrus control, the number of lymphocytes is very small or zero, with some identifiable epithelial cells (white arrow).

The characterization of the estrus stage of the animals was successfully determined by Pap smear; we determined the time of sexual inactivity between recurrent periods of estrus (the diestrus stage) as well as characterized the period of preparation for estrus (the proestrus stage) for all of the groups. Only the ovariectomized groups were permanently in the diestrus stage, indicating a successful ovariectomy (Figure 1).

### 2.2. Thyroid Stimulating Hormone Level Analysis

**Figure 2:**
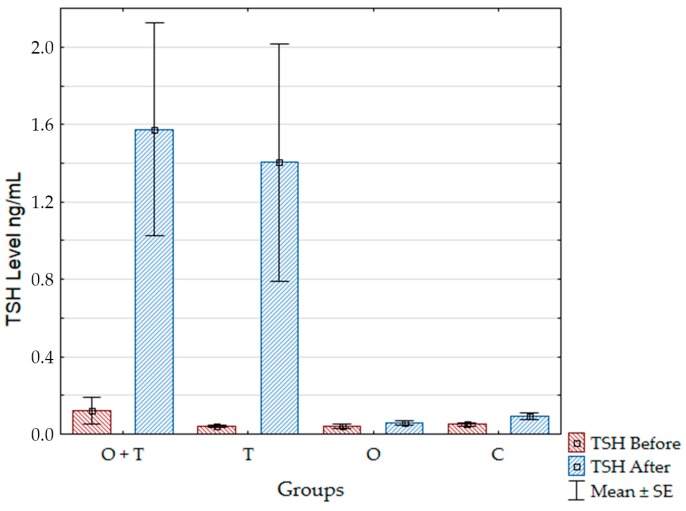
Thyroid Stimulating Hormone (TSH) values for the groups before and after the surgeries. SE: standard error; O + T: ovariectomized and thyroidectomized; O: ovariectomized; T: thyroidectomized; C: control.

We confirmed the effective removal of the thyroid gland by a detection of TSH values in the groups. We used the nonparametric statistical test Mann–Whitney and a *p* value of <0.05 was considered statistically significant. The TSH levels of the thyroidectomized (T) group and the ovariectomized and thyroidectomized (O + T) group were significantly higher after the surgeries compared to the TSH levels before the surgeries. The TSH of the ovariectomized (O) and the control (C) groups did not change after the surgery compared to samples taken before the surgery (Figure 2 and Table 1).

### 2.3. Flow Cytometry and Analysis of the Mononuclear Cells in the Bone Marrow

#### Endothelial Progenitor Cells

**Figure 3:**
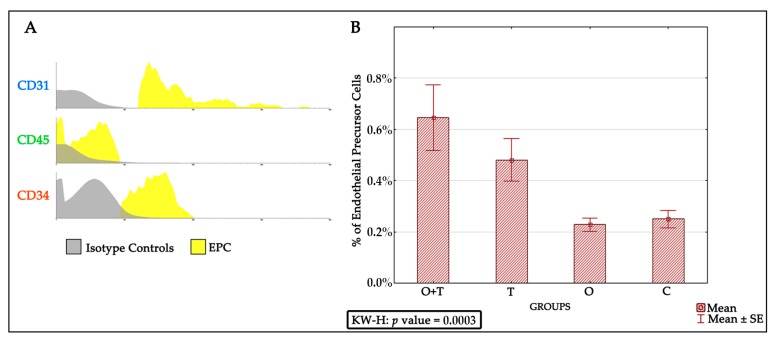
(**A**) Histogram of endothelial progenitor cells (EPCs) identified by CD31+, CD45−, and CD34+; and (**B**) Graph showing percentages of endothelial progenitor cells in each group and the result of the Kruskal-Wallis statistical test (KW-H), *p* value = 0.0003.

To confirm the identity of the endothelial progenitor cells, we identified typical endothelial progenitor cell phenotypes by positivity of CD31+, CD45−, and CD34+ in the thyroidectomy or/and ovariectomy groups as well as in the control animals (Figure 3A). Then, we were specifically interested to know if any of the analyzed groups would affect the proliferation of the endothelial progenitor cell population. We found an increased endothelial progenitor cell number in the thyroidectomized group. However, our analysis did not detect any change in the endothelial progenitor cell proliferation of the ovariectomized or control groups (Figure 3B and Table 2).

### 2.4. Precursor B Cells/Pro-B Cells

**Figure 4:**
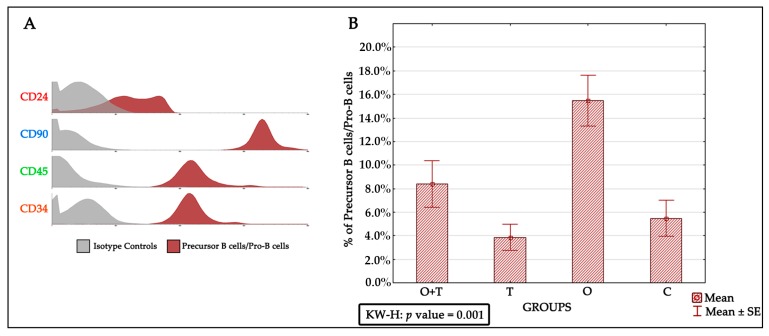
(**A**) Histogram indicating markers of precursor B cells/Pro-B cells in each studied group. These cells are characterized by appearances of CD24 +, CD90 +++, CD45 ++, and CD34 ++; and (**B**) Graph of the percent of precursor B/Pro-B cells in each group and the result of the Kruskal-Wallis statistical test, *p* value = 0.001.

We then thought to investigate the specific roles of ovarian or/and thyroid hormones in the regulation of precursor B cells’/Pro-B cells’ viability and multiplication. To study a number of precursor B cells/Pro-B cells after the removal of ovarian and/or thyroid hormones by ovariectomy and/or thyroidectomy, we first characterized these cells by the CD24+, CD90+, CD45+, and CD34+ phenotypes (Figure 4A). The analysis revealed an increase in precursor B cells/Pro-B cells number upon removal of ovarian hormones by ovariectomy (Figure 4B). This increase was not seen in the thyroidectomy group, which showed no significant difference from the control group (Figure 4B and Table 2). We were then interested to know if the removal of thyroid hormones would affect the increase in precursor B cells/Pro-B cells proliferation caused by a loss of ovarian hormones. When we removed both the thyroid and ovaries, we detected a slight (non-significant) increase in precursor B cells/Pro-B cells number (Figure 4B and Table 3).

### 2.5. Mesenchymal Stem Cells

**Figure 5:**
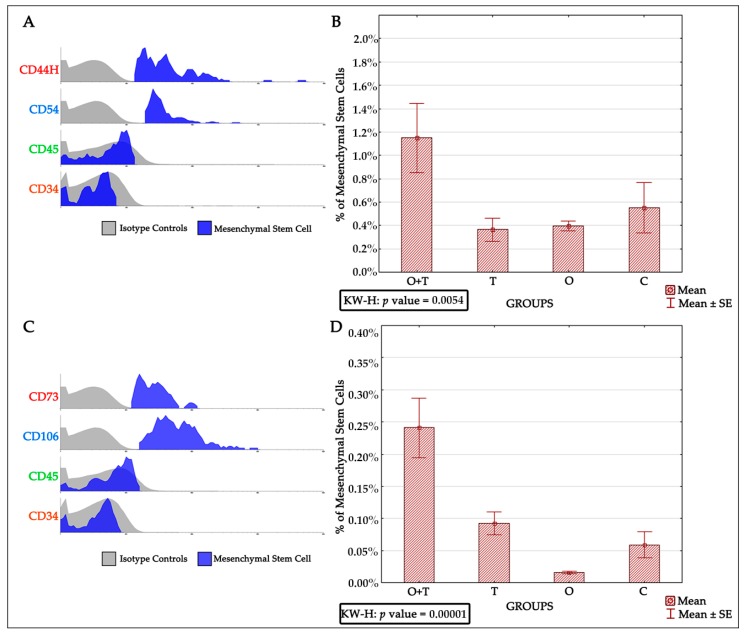
(**A**) Histogram of Mesenchymal Stem Cells (MSCs) characterized by CD44H++, CD54+++, CD45−, and CD34−; (**B**) Graph of the percentages of MSC number and the result of the Kruskal-Wallis statistical test, *p* value = 0.0054; (**C**) Histogram of the mesenchymal stem cells characterized by the presence of CD73+, CD106+, CD45−, and CD34−; (**D**) Graph of the percentages of MSC number and the result of the Kruskal-Wallis statistical test, *p* value = 0.00001.

We also wanted to know if the mesenchymal stem cells (MSCs) number was affected by the surgeries. MSCs were identified by the presence of the CD44H+, CD54+, CD73+, CD106+, CD34–, and CD45− phenotypes (Figure 5A,C). The removal of both ovarian and thyroid hormones caused a significant increase in MSCs number (Figure 5B,D, and Table 4 and Table 5). The ovariectomized group also had a significantly decreased MSCs number compared to the control group. This analysis was confirmed by assessing the phenotype markers CD73+/CD106+/CD45−/CD34− (Figure 5C and Table 5). Interestingly, no significant difference was observed in the thyroidectomized group when compared with the control group.

### 2.6. Bromodeoxyuridine Analysis

**Figure 6:**
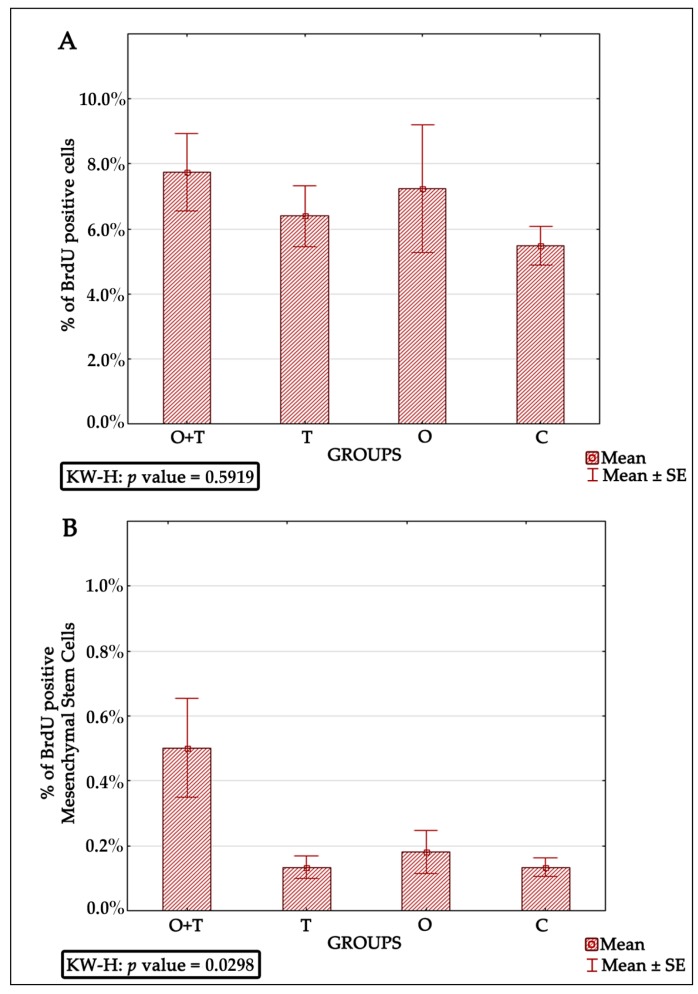
(**A**) Graph of the percent of the total mononuclear cell number assessed by identifying BRDU-positive cells. Kruskal-Wallis statistical test, *p* value = 0.05919; and (**B**) Graph of the percent of the MSCs with BRDU-positive cells and the result of the Kruskal-Wallis statistical test, *p* value = 0.0298. BRDU: Bromodeoxyuridine.

Our analysis of the total mononuclear cell proliferation did not reveal any significant difference between the studied groups (Figure 6A). Interestingly, an assessment of MSCs with BRDU+/CD106+/7AAD/CD34− revealed a significant increase in the proliferative activity of O + T group when compared to the control group (Figure 6B and Table 6). No other group showed a significant difference (Table 6) when compared to the control group.

## 3. Discussion

The use of BMDSCs has made a number of contributions to the study of regenerative medicine; however, the hormonal regulation of the biological activities of these cells is not fully understood. Experimental thyroidectomy and ovariectomy in animal models have led to many advances in the fields of cell biology, endocrinology, and metabolism, particularly with regard to studying the roles of thyroid and ovarian hormones in regulating mechanisms of cell survival, proliferation, differentiation, and programmed cell death. In the present study, we investigated endocrine-dependent events of bone marrow stem cell survival and proliferation upon the removal of the thyroid gland or/and ovaries. Some previous studies have studied the hormonal influence on cell biology by using thyroid inhibitors at the receptor and histone acetylation levels. Other studies have focused on disturbing mechanisms involved in thyroid hormone biosynthesis, cellular uptake, cellular transport, or nuclear action. These strategies are known to cause many problems, such as a disruption of oxygen consumption and peripheral metabolic abnormalities via unknown mechanisms [34,35,36]. Importantly, these methods were not efficient enough to analyze the effects of a complete loss of hormonal action. Therefore, to understand the role of a complete lack of ovarian or/and thyroid hormones in BMDSCs proliferation, we performed ovariectomies or/and thyroidectomies, respectively, in female Wistar rats.

The endothelial progenitor cells were identified via detecting for positivity of the CD31+, CD45−, and CD34+ phenotypes. We found an increased endothelial progenitor cell number in the thyroidectomized group. However, our analysis did not detect any change in the endothelial progenitor cell proliferation of the ovariectomized or the control groups. Because decreased estrogen affects total bone marrow mass, we asked if a decrease in ovarian hormones could block the stimulatory effect of thyroid removal on endothelial progenitor cells proliferation. Interestingly, our results indicate that the absence of ovarian hormone does not change the increased endothelial progenitor cell number caused by thyroid hormone removal (Figure 3).

Bone marrow EPCs are derived from CD34+ stem cells and undergo continuous differentiation into erythrocytes, thrombocytes, leukocytes, and mature endothelial cells [9,37]; however, the regulatory mechanisms are incompletely elucidated. EPCs are involved in neovascularization to improve cardiac function after cardiac ischemia [38]. In humans with ischemic hearts, the outcome highly correlates to the number of EPCs [39]. Thus, the modulation of EPC proliferation might be a useful therapeutic approach in the cardiovascular field.

Our results indicate significant thyroid hormonal action on EPCs number; however, other bone marrow signals that stimulate the proliferation of this lineage cannot be excluded. Further studies should investigate molecular pathways that govern the effect of thyroid hormones on endothelial progenitor cell proliferation. Thyroid hormone stimulates expression of its target genes through the thyroid receptor’s activation [40]. Transcription downstream of the thyroid hormones can be activated by the ligand-bound form of thyroid receptors. Previous reports have shown that the absence of thyroid hormone affects the proliferation of cancer cells via regulating the cell cycle gene [41]. Therefore, changes of EPCs number upon thyroid removal may involve the expression of thyroid hormone targets that possibly include genes governing cell division, such as cyclins and/or cyclin-dependent kinases. Our results provide a good basis for future studies investigating mechanisms that regulate thyroid hormone-dependent EPCs proliferation.

Previous studies have proposed various mechanisms that affect precursor B cells/Pro-B cells in aged animals. These include survival factors, altered differentiation, and impaired production of precursor B cells/Pro-B cells. However, our understanding of how cell proliferation and its mechanisms are regulated by hormones remains fragmentary. One of the major goals of this study was to understand the regulation of precursor B cells/Pro-B cells number in aged female Wistar rats. Earlier works have shown that the precursor B cells/Pro-B cells pool is decreased by ~fourfold in aged adults [36]; however, this decrease was postulated to be an effect of decreased function caused by the advanced age of many different body systems that impair the physiology of the aged animals. In addition, precursor B cells/Pro-B cells number in old mice has been proposed to be regulated by neighboring bone marrow stromal cells and soluble molecules [42,43,44]. We therefore sought to investigate the specific roles of ovarian or/and thyroid hormones in the regulation of precursor B cells’/Pro-B cells’ viability and multiplication. To study the proliferation of precursor B cells/Pro-B cells after the removal of ovarian and/or thyroid hormones by ovariectomy or/thyroidectomy, we first characterized these cells by the CD24+, CD90+, CD45+, and CD34+ phenotypes. Interestingly, our analysis revealed an increase in precursor B cells/Pro-B cells number upon the removal of ovarian hormones by ovariectomy. This increase was not seen in the thyroidectomy group, which showed no significant difference from the control group. We were then interested to know if the removal of thyroid hormone would affect the increase in precursor B cells/Pro-B cells proliferation caused by a loss of ovarian hormones. When we removed both the thyroid and ovaries, we detected a slight (non-significant) increase in precursor B cells/Pro-B cells number (Figure 4). This suggests that a loss of thyroid hormone does not affect the decrease in precursor B cells/Pro-B cells number caused by a lack of ovarian hormone.

Previous results have shown an association between certain regulatory genes and the developmental sequence of precursor B cells/Pro-B cells [43,44]. For instance, precursor B cells/Pro-B cells production is greatly reduced when there is a decreased amount of mRNA of the recombination activating genes 1 and 2 in aged bone marrow [42,45]. Moreover, signals from stromal cells have been shown to potentiate the effect of other gene groups, such as the interleukin 7 receptor, that modulate the proliferation and differentiation of developing B cells [46,47,48,49,50]. These events may require direct cell communication with stromal cells and biological responses to signaling growth factors [51,52]. Future studies may investigate if ovarian hormones control precursor B cells/Pro-B cells number through these molecular mechanisms. Identification of pathways downstream of ovarian hormones may shed new light towards dissecting the genetic programs controlling cell proliferation of the developing B cell lineage. In addition, determination of the pro-B cells that receive hormonal signals will improve our understanding of the physiology of the bone marrow of aging mammals.

The majority of immature bone marrow precursor B cells/Pro-B cells undergo significant proliferation to reach the optimum number [53,54,55]. It is important to note that some B lineage precursors may die to balance cell proliferation in the bone marrow. Therefore, the production of this lineage is accompanied by growth, differentiation, and apoptosis [55,56,57,58,59]. A disturbance of these processes might be affected by ovarian hormone action. Since we do not see a difference in the number of dying precursor B cell/Pro-B cells, we suggest that the ovarian control of Pro-B cells mainly affects cell proliferation.

Bone marrow-derived mesenchymal stem cells have been reported to be very efficient in regenerative medicine due to their multi-lineage differentiation character [60,61] and high potential to repair tissues [62,63,64]. However, a critical obstacle is how their cell number is controlled [65]. Thus, successful mesenchymal cell therapy requires better elucidation of the regulatory mechanisms controlling the proliferation of these cells. Our analysis also evaluated the roles of thyroid or/and ovarian hormones in the proliferation of mesenchymal bone marrow stem cells. Adult female rats from the thyroidectomy and/or ovariectomy groups were analyzed for changes in mesenchymal cell number and compared with control animals. MSCs were identified by the presence of the CD44H+, CD54+, CD73+, CD106+, CD45−, and CD34− phenotypes (Figure 5). Our BRDU analysis revealed no effect of thyroidectomy on the number of mesenchymal bone marrow cells when compared to the control group. Interestingly, the removal of both ovaries and thyroid glands caused a significant increase in mesenchymal stem cell number in the bone marrow in comparison to the control group (Figure 6). Earlier works have shown that MSCs can maintain their multipotency ability and proliferate rapidly at considerably low densities, but this expansion may not generate adequate numbers of mesenchymal cells for therapeutic applications [63,64,66,67]. Our results of increased MSCs upon the removal of both thyroid and ovarian hormones indicate a necessity to generate highly proliferating mesenchymal cells, and may thus enhance efficient therapeutic regeneration.

**Figure 7:**
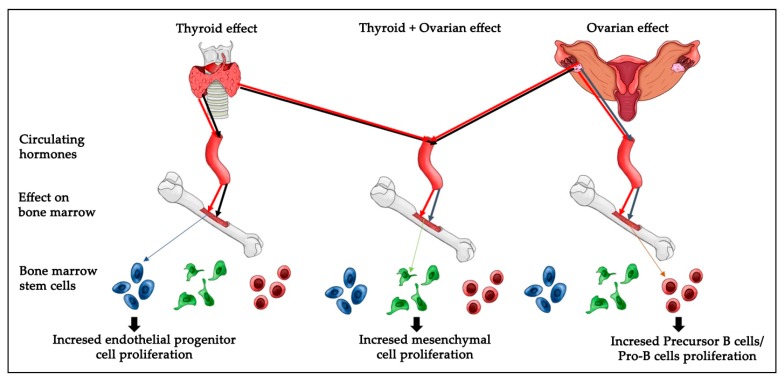
Proposed model of ovarian or/and thyroid hormonal effects on bone marrow stem cells. Ovariectomy leads to increased Pro-B cell proliferation (black arrows). Thyroidectomy leads to increased endothelial progenitor cell proliferation (black arrows). The removal of the ovaries and thyroid leads to increased mesenchymal cell proliferation (black arrows). Black arrows illustrate the hormonal loss and its effect on cell proliferation. Red arrows indicate the normal hormonal situation (no effect on normal cell proliferation).

Cell viability and proliferation are major concerns in the field of stem cell biology as well as in therapeutic applications. It is critical to understand the in vivoresponse of BMDSCs, as successful models widely used in translational research, to physiologic and pathologic endocrine alterations. Future studies could investigate if monitoring the conditions of patients receiving stem cell therapy is related to thyroid and ovarian hormones homeostasis. Overall, the strength of our findings is that they were performed in an in vivo model. In summary, the present results reveal that hypothyroidism causes a significant increase in endothelial progenitor cells, whereas a loss of ovarian hormones significantly increased the numbers of precursor B cells/Pro-B cells and decreased the bone marrow mesenchymal cell number. The removal of both ovaries and thyroid glands caused a significant increase in mesenchymal stem cells number (Figure 7). However, it is not clear which molecular pathways regulate the cellular response to hormonal loss in each condition and which amount of each hormone is sufficient to induce specific changes in cell number. Thus, further research is important to elucidate the molecular and cellular biological mechanisms regulating thyroid or/and ovarian hormonal effects on BMDSCs number.

## 4. Materials and Methods

### 4.1. Animals and Surgical Procedures

A total of forty female Wistar rats, aged approximately three months, with weights ranging from 250 to 350 g were used. They were separated into four distinct groups, namely (C) control, animals which were not subjected to surgery, (O) ovariectomized, (T) thyroidectomized, and (O + T). The rats were housed in ventilated cages under standard laboratory conditions as well as standard temperature (20 ± 2 °C), operating on a 12-h dark/light condition with a relative humidity of 30–70%. Three to four animals were placed in a single cage. The cages were properly separated, identified with badges for each individual animal, and containedthe rat group number and the date of surgery. The rats had free access to water and standard animal food. The thyroidectomized rats received adequate calcium treatment. All rats that underwent surgery were anesthetized by administering intramuscular ketamine (50 mg/kg) and xylazine (10 mg/kg), then an ovariectomy or/and a thyroidectomy was/were performed. The protocol of these studies was approved by an institutional committee for laboratory animal control (CEUA-007-09, 11 05 2009), and it was in agreement with the guidelines for the care and use of laboratory animals published by the ARRIVE guidelines [68].

### 4.2. Measurement of Thyroid Stimulating Hormone Level

To confirm the loss of thyroid hormone in the thyroidectomized animals, we obtained TSH levels of various blood samples. Thirty days after the surgical procedures on the thyroid gland and ovaries, a cardiac puncture needle 25 × 07 mm was used under an anesthetic condition (ketamine 50 mg/kg) to withdraw 3 mL of blood for measurement of TSH (electrochemiluminescence method autoanalyzer/Elecsys/Roche). Then, three milliliters of 0.9% saline solution were administered into the peritoneal cavity of the animals for volume reposition to avoid dehydration.

### 4.3. Pap Test

To confirm the diestrus stage, smears from all rats of all groups were obtained, examined by Papanicolaou staining, and analyzed as described by Long Evans (1922) and Mandl (1951). The protocol used for staining was modified from Hubscher et al. (2005) [69].

### 4.4. Isolation of Bone Marrow-Derived Stem Cells from the Bone Marrow

After puncturing the iliac crest, the collected bone marrow blood was transferred to a 15 mL flask, and mixed with sterile phosphate buffered saline (PBS) [1]. The tubes were centrifuged for 5 min at 1400 rpm (350 G). After completion of the centrifugation, the supernatant was removed and mixed with 7 mL Dulbecco’s Modified Eagle’s Medium (DMEM). Seven milliliters (7 mL) of solution were then transferred to a 15 mL tube containing 3 mL density gradient Ficoll-Hypaque (density = 1.077 g/mL). The tube was centrifuged for 40 min at 1500 rpm (400 G). After centrifugation and the appearance of a characteristic ring at the interface, the solution was transferred into another 15 mL tube, followed by adding up to 10 mL sterile PBS and centrifugation for 5 min at 1500 rpm (400 G). The supernatant was then discarded and the pellet was suspended in 1 mL of PBS/albumin 5%. Cell counting was done in a Neubauer chamber, and cell viability was tested using the Trypan Blue vital dye.

### 4.5. Immunophenotyping and Flow Cytometry

This procedure confirmed that the bone marrow cells had maintained their phenotypic characteristics, as well as enabled cell quantification after the surgeries. In brief, 100 mL of isolated cells were put in 12 × 75 mm falcon tubes for immunophenotyping and cytometry. The cells were maintained in PBS/5% albumin, (nine tubes per rat). Antibody labeling was used to identify the various cell types. After homogenization, the cells were incubated for 15 min with the primary antibodies. They were then washed with 400 μL PBS/5% albumin and centrifuged (1400 rpm (350 G), 5 min). The supernatant was discarded, and the cells were suspended in 100 mL PBS/5% albumin and incubated with the FITC-conjugated antibody (Rat Anti-Mouse IgG1-3 μL) for 15 min in the dark. After incubation, the cells were washed with 400 μL PBS/albumin 5% and centrifuged (1400 rpm (350 G), 5 min), then the supernatant was discarded and the cells were suspended in 100 mL PBS/5% albumin. Monoclonal antibodies were used following the incubations and washing procedure mentioned above. Finally, the cells were suspended in 250 μL PBS/5% albumin and analyzed by flow cytometer (Becton Dickinson, San Jose, CA, USA) (Appendix A).

### 4.6. Bromodeoxyuridine Incorporation

BRDU-incorporated cells (representing the S-phase of the cell cycle) were analyzed with the BrdU Staining Kit Protocol Flow (Becton Dickinson, San Jose, CA, USA), according to the manufacturer’s recommended instructions. In brief, cells were surface stained then fixed and permeabilized by BD Cytofix/Cytoperm Buffer. After one hour of incubation with 30% of Dnase (*v/v*) (in PBS buffer at 37 °C), the cells were stained with anti-BRDU-FITC-conjugated antibody. The fluorescent DNA-binding stain 7-aminoactinomycin (7-AAD) was added to the samples right before the flow cytometry procedure.

## Figures and Tables

**Table 1:** Mean values ± standard error of the Thyroid Stimulating Hormone (TSH) levels before and after the surgeries of the studied groups and Mann-Whitney statistical test *p* values.

Groups	Mean before Surgery ± SE	Mean after Surgery ± SE	(*p* < 0.05) *
O + T	0.122 ± 0.069	1.575 ± 0.551	0.035693
T	0.040 ± 0.007	1.403 ± 0.613	0.011719
O	0.041 ± 0.009	0.056± 0.011	0.067890
C	0.051± 0.008	0.092 ± 0.017	0.142214

* Mann-Whitney statistical test was used and *p* values <0.05 were considered statistically significant.Thyroidectomized (T); ovariectomized and thyroidectomized (O + T); ovariectomized (O); control (C).

**Table 2:** Mann-Whitney post-hoc statistical test for the endothelial progenitor cells.

Groups	(*p* < 0.05) *
O + T vs. C	0.005129
T vs. C	0.010113
O vs. C	0.850107

* Mann-Whitney statistical test was used and *p* values <0.05 were considered statistically significant. Thyroidectomized (T); ovariectomized and thyroidectomized (O + T); ovariectomized (O); control (C).

**Table 3:** Mann-Whitney post-hoc statistical test for the precursor B cells/Pro-B cells.

Groups	(*p* < 0.05) *
O + T vs. C	0.270345
T vs. C	0.307435
O vs. C	0.002202

* Mann-Whitney statistical test was used and *p* values <0.05 were considered statistically significant. Thyroidectomized (T); ovariectomized and thyroidectomized (O + T); ovariectomized (O); control (C).

**Table 4:** Mann-Whitney post-hoc statistical test for the MSCs (CD44H/CD54/CD45/CD34).

Groups	(*p* < 0.05) *
O + T vs. C	0.022244
T vs. C	1.000000
O vs. C	0.384674

* Mann-Whitney statistical test was used and *p* values < 0.05 were considered statistically significant. Thyroidectomized (T), ovariectomized and thyroidectomized (O + T), ovariectomized (O), control (C).

**Table 5:** Mann-Whitney post-hoc statistical test for the MSCs (CD73/CD106/CD45/CD34).

Groups	(*p* < 0.05) *
O + T vs. C	0.001234
T vs. C	0.079180
O vs. C	0.000670

* Mann-Whitney statistical test was used and *p* values < 0.05 were considered statistically significant.Thyroidectomized (T), ovariectomized and thyroidectomized (O + T), ovariectomized (O), control (C).

**Table 6:** Mann-Whitney post-hoc statistical testfor the BRDU-positive MSCs.

Groups	(*p* < 0.05) *
O + T vs. C	0.021684
T vs. C	0.691102
O vs. C	0.870282

* Mann-Whitney statistical test was used and *p* values < 0.05 were considered statistically significant. Thyroidectomized (T), ovariectomized and thyroidectomized (O + T), ovariectomized (O), control (C).

## Abbreviations

BMDSCs	Bone marrow-derived stem cells
TSH	Thyroid-stimulating hormone
EPCs	Endothelial progenitor cells
MSCs	Mesenchymal stem cells
T4	3,5,3′,5′-Tetraiodo-l-thyronine
T3	3,5,3′-Triiodothyronine
BRDU	Bromodeoxyuridine
O + T	Ovariectomized and thyroidectomized
T	Thyroidectomized
O	Ovariectomized
C	Control
PBS	Phosphate buffered saline
DMEM	Dulbecco’s modified eagle’s medium
7-AAD	7-Aminoactinomycin

## Appendix A

**Table A1:** Panel of the flow cytometry configuration.

Tube	(FITC/PE/PerCP/APC)
1	Isotypic Control
2	Cell Control
3	/CD31/CD45/CD34
4	CD24/CD90/CD45/CD34
5	CD44H/CD54/CD45/CD34
6	CD73/CD106/CD45/CD34
7	BRDU/-/7AAD/-
8	BRDU/CD106 /7AAD/CD34
